# Effects of Omega-3 Polyunsaturated Fatty Acids and Their Metabolites on Haemostasis—Current Perspectives in Cardiovascular Disease

**DOI:** 10.3390/ijms22052394

**Published:** 2021-02-27

**Authors:** Jacek Golanski, Patrycja Szymanska, Marcin Rozalski

**Affiliations:** Department of Haemostasis and Haemostatic Disorders, Chair of Biomedical Sciences, Faculty of Health Sciences, Medical University of Lodz, Mazowiecka 6/8, 92-235 Lodz, Poland; jacek.golanski@umed.lodz.pl (J.G.); patrycja.szymanska@stud.umed.lodz.pl (P.S.)

**Keywords:** omega-3, PUFA, DHA, EPA, haemostasis, platelet, endothelium, cardiovascular, COVID-19

## Abstract

The beneficial effects of long-chain polyunsaturated omega-3 fatty acids (omega-3 PUFAs) in cardioprotection are widely known and generally accepted. In this literature review, we have focused on the known and postulated mechanisms of action of omega-3 PUFAs and their metabolites on various components of the haemostatic system, in particular on blood platelets and endothelium. We have also made an attempt to provide a comprehensive review of epidemiological studies with particular regard to clinical trials. Notably, the results of these studies are contradictory, and some of them failed to report the beneficial effects of taking or supplementing omega-3 PUFAs in the diet. A potential explanation, in our opinion, could be the need to use higher doses of omega-3 PUFAs and a proper ratio of omega-3 and omega-6 PUFAs. An additional problem which is difficult to solve is the use of a proper neutral placebo for interventional studies. Despite some controversies regarding the beneficial effects of supplementation of omega-3 PUFAs in cardiovascular disease, our review suggests that a promising aspect of future studies and applications is to focus on the anti-thrombotic properties of these compounds. An argument supporting this assumption is the recent use of omega-3 PUFAs as a supporting tool for the treatment of COVID-19 complications.

## 1. Introduction

The beneficial effects of long-chain polyunsaturated omega-3 fatty acids (omega-3 PUFAs) are widely accepted based on the evidence that supplementation with omega-3 PUFAs enhances cardioprotection in patients at cardiovascular risk or with cardiovascular disease (CVD) [1,2]. It is known that CVD results, among other causes, from the hyperreactivity of blood platelets, their enhanced aggregation and dysfunction of the endothelium [3,4,5]. The mechanisms of action of omega-3 PUFAs on haemostasis are complex, and their contribution to final outcomes has not been fully elucidated [6,7,8].

Haemostasis is defined as a balanced set of defence mechanisms of an organism allowing it to maintain blood circulation and the prevention of blood loss in the case of the integrity of blood vessels being broken. Additionally, the haemostatic system plays a role in the regulation of function of the vascular bed and immune response. Major components of haemostatic system are the vessel wall (including endothelium), blood platelets, coagulation and fibrinolytic systems [9].

There is increasing scientific evidence supporting a beneficial role of polyunsaturated fatty acids (PUFAs) in the prevention and treatment of CVD. Particular cardioprotective properties are attributed to PUFAs from the omega-3 group due to their anti-aggregatory and anti-inflammatory properties [10,11,12,13]. Current studies concerning the effects of omega-3 PUFAs on haemostasis focus predominately on blood platelets and endothelial cells. This is understandable, as according to the cell theory of the regulation of haemostasis, both platelets and endothelial cells are largely responsible for the pathophysiology of cardiovascular disease. Omega-3 PUFAs are known to affect both platelets and the endothelium. Thus, the inhibition of the procoagulant activity of both cell types is believed to be one of the most important mechanisms in cardioprotection [2].

In the case of patients with CVD, it is recommended to increase the supply of eicosapentaenoic acid (EPA) and docosahexaenoic acid (DHA), which is intended to enhance the classical pharmacological treatment. As an example, the American Heart Association (AHA) recommends a supplementation of EPA and DHA in the form of fish oil in a dose of 2–4 g/day for patients with hypertriglyceridemia [14]. For all CVD patients, it is extremely important that the number of capsules intended to be consumed daily should reflect the recommended dose of EPA and DHA. Obviously, the manufacturers are obliged to provide information on the actual content of fatty acids contained in the formulation. However, a certain discrepancy sometimes exists between the declared and actual content. There are reports suggesting that such discrepancies are greater for DHA than for EPA [15]. Additionally, do Vale et al. described methodological problems associated with investigating the cardiovascular effects of omega-3 PUFAs. These problems are mainly due to the use of inappropriate (active) placebos in studies related to omega-3 PUFAs, which highlights the importance of choosing a truly inert placebo to maintain the high reliability of randomized clinical trials [16].

## 2. Metabolism and Mechanism of Action of Omega-3 PUFAs

Omega-3 and omega-6 PUFAs are components of phospholipids, which are constituents of cell membranes. Due to the catalytic action of phospholipase A_2_ (PLA_2_), these fatty acids are released from the membrane and become precursors of eicosanoids, such as prostaglandins, thromboxanes, leukotrienes or lipoxins, with a plethora of biological effects [17].

**Figure 1:**
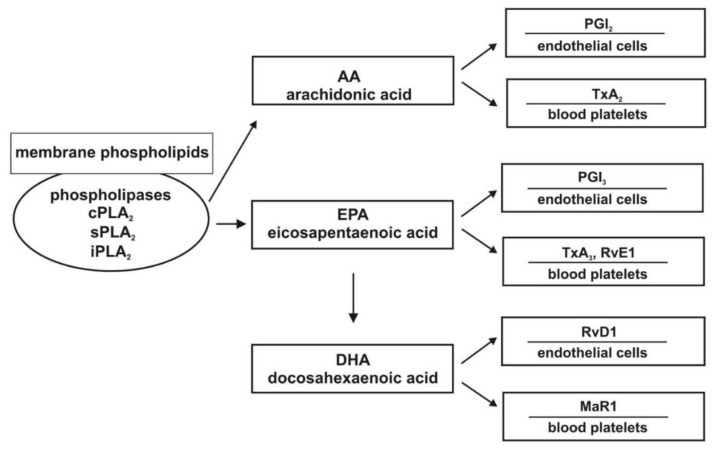
Association between metabolites of unsaturated fatty acids and platelets, as well as endothelial cells. Abbreviations: PLA_2_: Phospholipase A_2_; MaR1: Maresin 1; PGI_2_: Prostacyclin; TXA_2_, TXA_3_: Thromboxanes; RvE1: Resolvin E1; RvD1: Resolvin D1; AA: Arachidonic acid; DHA: Docosahexaenoic acid; EPA: Eicosapentaenoic acid.

An important member of the omega-6 PUFAs is linoleic acid (LA), which is the precursor of arachidonic acid (AA) and characterized by pro-inflammatory properties. By contrast, ALA is a major fatty acid from the omega-3 group and is a precursor of EPA, which is a substrate for DHA. These fatty acids are characterized by anti-inflammatory properties [18,19,20]. The metabolic pathway of AA by cyclooxygenase (COX) results in the production of eicosanoids. The synthesis of pro-aggregatory thromboxane A_2_ (TXA_2_) takes place in blood platelets, whereas prostacyclin I_2_ (PGI_2_) is produced in the endothelium, with an antagonistic effect. Thromboxane A_3_ (TXA_3_) synthesized in platelets exhibits only slight pro-aggregatory activity. In turn, prostaglandin I_3_ (PGI_3_) produced in the endothelium possesses anti-aggregatory properties. TXA_3_ and PGI_3_ are products of the metabolism of EPA [17,18,20]. The anti-aggregatory effects of prostaglandins, especially PGI_2_ that has originated from AA, results from binding to the Gs protein-coupled receptor, which leads to an increase in intracellular cAMP concentration and the reduction of the synthesis of TXA_2_ [21,22,23,24]. Figure 1 presents the simplified metabolic pathways of AA, EPA, and DHA.

## 3. Omega-3 PUFAs and Blood Platelets

EPA and DHA play an important role in maintaining physiological haemostasis and, due to their antiplatelet and anticoagulant properties, reduce the risk of occurrence of cardiovascular events. EPA and DHA are incorporated into the cell membrane, altering its fluidity and thus regulating haemostasis, including thrombin generation [25,26,27]. EPA and DHA exert inhibitory effects on platelet aggregation. LA and ALA, in turn, compete with each other for enzymes (desaturases and elongases) essential for further metabolic processes. A higher dietary intake of ALA in comparison with LA leads to an enhanced synthesis of EPA and DHA metabolites, which results in the higher production of TXA_3_ than the pro-aggregatory TXA_2_. The possible effect is also an inhibition of cyclooxygenases as well as a direct antagonistic influence on the receptor for prostaglandin H_2_-TXA_2_. Prostaglandin H_2_ is a precursor of other prostaglandins and thromboxanes [9].

**Figure 2:**
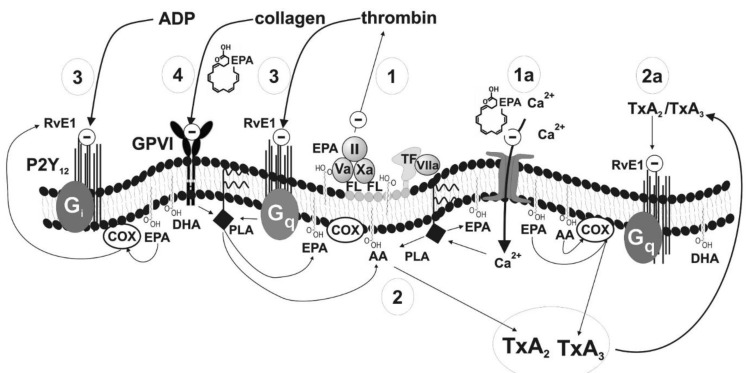
Mechanism of antiplatelet effects of eicosapentaenoic acid (EPA), docosahexaenoic acid (DHA), and their metabolites. Abbreviations: II-Va-Xa: Factors of prothrombin complex; AA: Arachidonic acid; COX: Cyclooxygenase; FL: Membrane phospholipids; GPVI: Collagen receptor; Giq: G proteins; PLA: Phospholipase; P2Y12: ADP receptor; TF: Tissue factor; TXA_2_, TXA_3_: Thromboxanes; RvE1: Resolvin E1.

The presented mechanisms of the effects of omega-3 PUFAs (Figure 2) are described briefly below on the basis of published data (Table 1). Summarizing the information given in the table, it is widely accepted that blood platelets play an important role in haemostasis not only due to activation and aggregation but also as they are involved in thrombin generation. Thrombin is a strong platelet agonist but is also involved directly in thrombus formation. The excessive activation and aggregation of platelets can result in adverse cardiovascular events such as myocardial infarction or ischaemic stroke. Omega-3 PUFAs could enhance conventional therapy via the regulation of blood platelet function [28].

Special attention should be paid to resolvins, which are so-called pro-resolving mediators (also termed specialized pro-resolving mediators (SPMs)) with anti-inflammatory properties. The substrates for their production are both EPA (resolvins E) and DHA (resolvins D). They contribute to the reduction of acute inflammatory response as well as inhibit thromboxane-induced platelet aggregation [29,30]. Additionally, DHA is a substrate for the generation of anti-inflammatory agents such as marisins. These substances, together with resolvins D, are classified as docasonoids [18,20]. In a recent publication, Yamaguchi et al. demonstrated that DHA 12-LOX oxylipins inhibited platelet reactivity in a glycoprotein VI-dependent manner due to the activation of protein kinase A. DHA and its 12-LOX-derived oxylipins also affected collagen-induced platelet aggregation. DHA and its oxylipins, 11-HDHA and 14-HDHA, were shown to regulate agonist-induced platelet aggregation [8].

## 4. Omega-3 PUFAs and Endothelium

**Figure 3:**
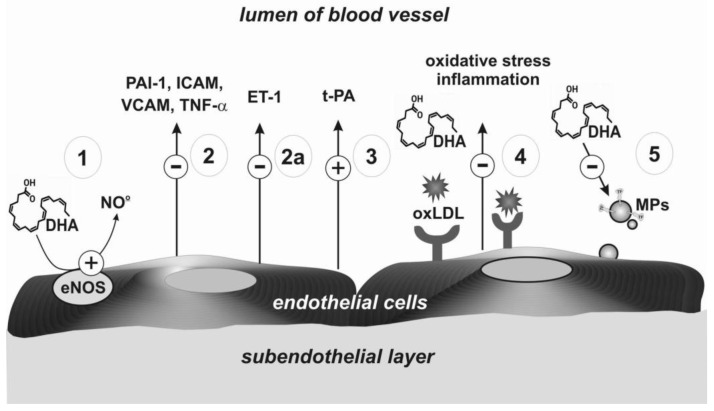
Mechanisms of endothelium regulation mediated by EPA, DHA, and their metabolites. Abbreviations: ET-1: Endothelin 1; eNOS: Endothelial synthase of nitric oxide; ICAM: Intercellular adhesion molecule; MPs: Microparticles; oxLDL: Oxidized low-density lipoproteins; NO: Nitric oxide; PAI-1: Plasminogen activator inhibitor 1; t-PA: Tissue plasminogen activator; TNF-α: Tumour necrosis factor-α; VCAM: Vascular cell adhesion molecule.

The beneficial effects of AA, EPA, and DHA metabolites have also been observed on endothelial cells. This is mainly due to the stimulating nitric oxide (NO) production [37,38]. The mechanisms are presented in Figure 3 and described in Table 2. The data summarised in Table 2 suggest that omega-3 PUFAs demonstrate a protective effect on the endothelium via both the NO synthesis and antioxidant, as well as anti-inflammatory properties. Altogether, this is important for the prevention and limiting of the development of atherosclerosis, and in consequence in the prevention of myocardial infarction and ischaemic stroke [38].

## 5. Epidemiological Studies and Clinical Trials

In the literature, there are many publications reporting the effects of omega-3 PUFAs supplementation in cardiovascular disease. The use of omega-3 PUFAs in the prevention of cardiac incidents (both primary and secondary) was addressed by relatively novel randomized clinical trials such as: JELIS [46], VITAL [47], STRENGTH [48], and ASCEND [49]. Other studies investigated not only the benefits of supplementation but also the potential risks [16,50,51]. However, since in this review we focus mainly on the effects of omega-3 PUFAs on haemostasis, below we will describe epidemiological studies concerning this particular issue.

The hypothesis that omega-3 PUFAs can affect haemostasis originated on the basis of the observations of a population of Inuits—native inhabitants of Greenland [52]—in whom a reduced reactivity of blood platelets, longer bleeding time, and a lowered ratio of thromboxanes to anti-aggregatory prostacyclins was found. The typical diet of Inuits is known for its high consumption of long-chain omega-3 PUFAs, especially DHA and EPA derived from fatty marine fish. The above-described hypothesis was confirmed in clinical studies [53].

In the work of McEwen et al., CVD patients and healthy controls were administered a 4-week supplementation of omega-3 PUFAs (640 mg/day). Interestingly, the reactivity and activation of blood platelets were lowered in healthy donors but not in CVD patients. The conclusion, and the suggestion of the authors, is the need to use higher doses of omega-3 PUFAs in these patients [23]. The concept of the application of higher doses of omega-3 PUFAs has been confirmed in a paper by Li et al. They reported that a supplementation of EPA as high as 6 g/day is required to reduce platelet adhesion [54].

Several studies carried out in humans have demonstrated that omega-3 PUFAs have antiplatelet properties. A meta-analysis of 15 randomised controlled trials provided evidence that omega-3 PUFAs inhibited blood platelet aggregation [22]. Marine omega-3 PUFAs were also observed to overcome aspirin resistance (acetylsalicylic acid (ASA)) [28,55]. In studies on laboratory animals, it was demonstrated that ASA enhanced the antiplatelet effect of fish oil [56]. In healthy but overweight men, 3 g of omega-3 PUFAs administered for 4 weeks lowered the concentrations of fibrinogen, thrombin, and factor V. Interestingly, these effects occurred mainly in subjects who were carriers of alpha-chain fibrinogen polymorphism [57]. It was found that, in a double-blind placebo-controlled study in 30 healthy subjects taking 2.52 g/day of omega-3 PUFAs as compared with 1.26 g/day for 5 weeks, the group with a higher dose of omega-3 PUFAs displayed decreased plasma viscosity, red blood cell rigidity, and systolic blood pressure [58]. In the study performed on healthy adults, it was reported that fish oil (equivalent to 6 g of EPA/day), but not vegetable oil, significantly reduced platelet adhesion [54]. In another work, it was found that supplementation with 3.6 g of omega-3 PUFAs from fish oil lowered platelet aggregation, whereas 25 g of soy lecithin (providing 1.5 g omega-6 and 0.5 g omega-3 PUFAs) enhanced platelet reactivity [59]. The omega-6/omega-3 ratio in platelets is also positively correlated with platelet adhesion both in resting platelets, as well as after the activation of platelets by agonists such as ADP and thrombin. Therefore, it seems that higher doses of marine omega-3 PUFAs result in more effective antithrombotic benefits.

In the trial REDUCE-IT (Reduction of Cardiovascular Events with Icosapent Ethyl-Intervention Trial), 8179 patients with hypertriglyceridemia and co-existing diabetes mellitus or additional cardiovascular disorders were studied. The patients were treated with statins and randomized in a double-blind manner to either icosapent ethyl at 4 g/day (2 g twice daily with meals) or placebo groups [60]. Despite the fact that this trial did not analyse the effect of icosapentethyl on haemostasis, the results of this study could be treated as a suggestion of the need to use high doses of EPA derivatives. In other studies, a reduction of the incidence of adverse cardio-vascular events of 25% was observed [6,60,61]. On the other hand, in another double-blind trial on patients with hypertension and diabetes, it was found that only DHA significantly reduced platelet aggregation induced with pro-coagulatory factors [62].

It is noteworthy that the proper ratio of omega-6 and omega-3 PUFAs in a diet is also very important [63]. It was demonstrated that a high omega-6/omega-3 ratio enhanced platelet aggregation. It seems that omega-6 PUFAs have a pro-coagulatory and pro-inflammatory activity. LA, belonging to the omega-6 group, lowers low-density lipoprotein (LDL) levels. On the other hand, it increases the predisposition of LDL to oxidation. This is a dangerous process, leading to a higher risk of coronary artery disease. It is believed that omega-3 and omega-6 PUFAs should be balanced when they are taken in a diet in a ratio of about 1 to 1 [64].

Supplementation with omega-3 and omega-6 PUFAs is used to limit the progression of cardiovascular disease and is applied both in primary as well as secondary prevention [28]. Experimental studies also suggest that EPA and DHA inhibit platelet aggregation in vitro. However, DHA exerts a stronger effect on the platelet function [28]. Despite the fact that epidemiological data suggest a diet rich in omega-3 PUFAs is effective in CVD prevention, due to the anti-inflammatory and anti-platelet activity [28], some authors question the effectiveness of such a supplementation [50,65,66]. The lack of unambiguous recommendations regarding the supplementation results also from the controversial results of studies investigating the effect of omega-3 PUFAs on haemostasis. The review of the literature reveals that not all the publications confirm the beneficial effects of omega-3 PUFAs on haemostasis. Poreba et al. investigated the impact of the daily supplementation of 2 g omega-3 PUFAs in patients with advanced atherosclerosis and diabetes on platelet function and fibrinolysis, as well as thrombus formation. The study did not yield any positive results [67]. Similarly, this effect was not demonstrated in healthy volunteers [68].

The studies performed by our group demonstrated that a higher consumption of omega-3 PUFAs had a significant inhibitory effect on inflammatory markers, whereas blood platelet reactivity was not affected [69,70]. However, the lack of a significant effect of omega-3 PUFAs on the platelet function in these studies can be explained by the insufficient consumption of omega-3 PUFAs (EPA + DHA below 1 g/day). This hypothesis is in accordance with the results of Sing et al., who reported that supplementation of EPA and DHA in a dose of 1.24 g/day inhibited the inflammatory response, which manifested as a reduced level of C-reactive protein in serum [71]. Assuming the hypothesis that an anti-aggregatory effect of omega-3 PUFAs is mediated also via a mechanism mediated by resolvins, it seems that a diet or a supplementation should contain higher doses than those described above for the substrates of resolvins, i.e., EPA and DHA. With regards to the anti-aggregatory potential, the most interesting resolvin appears to be resolvin E1 (RvE1) [29,72]. RvE1 is a metabolite of EPA and was found to inhibit ADP-induced platelet aggregation [35].

The diet supplementation of patients suffering from coronary artery disease with omega-3 PUFAs in a dose of 2 g/day was found to have significant effects on the endothelium function or the secretion of tissue plasminogen activator (t-PA), which plays a role in a plasmin production, limiting thrombus formation [43]. In another randomized study, patients with peripheral artery disease were supplemented with either fish oil for 6 weeks (with a daily dose of EPA and DPA of 850–882 mg) or a placebo. The result was that no significant effect of this supplementation was observed with respect to the blood platelet activity, endothelium function, as well as inflammatory markers [73]. He et al. evaluated the influence of the consumption of fried and unfried fish as well as the consumption of omega-3 PUFAs on the endothelium function and inflammatory marker levels. It was found that the intake of omega-3 PUFAs was associated with a lower level of pro-inflammatory interleukin-6 (IL-6), irrespective of the remaining parameters evaluated in the patients. On the other hand, lower levels of C-reactive protein (CRP) and IL-6 were associated with the consumption of unfried fish, whereas the consumption of the fried fish resulted in a decrease of intercellular adhesion molecule (ICAM) expression. Therefore, it seems that the consumption of fish and omega-3 PUFAs leads to a cardioprotective effect resulting from the influence on the endothelium, as well as to a lowering of the level of inflammatory markers and expression of adhesive proteins [37].

The normal function of the vascular endothelium plays an important role in the prevention of arterial disorders such as atherosclerosis. A dysfunction of the endothelium is associated with an impaired generation of NO, which normally exerts a vasodilatory effect. The protective effects of statins—drugs applied to treat dyslipidaemia as well as EPA—were demonstrated in [74]. In this study, the dual effect of statins (a metabolite of atorvastatin (ATM)) and EPA was assessed on the condition of an endothelium subjected to oxidative stress. It was found that the combined therapy yielded more beneficial results in comparison to ATM or EPA alone. Moreover, the same effect was not observed when EPA was replaced by DHA or when using other drugs which lower triglycerides levels, e.g., fenofibrate or gemfibrozil. This finding seems to be of special importance for disorders in which endothelium dysfunction leads to the development of cardiovascular disease [74].

The list of epidemiological studies and clinical trials, including author, year, type of study, source and dose of omega-3 PUFAs, duration of supplementation or observation, outcome, and commentary is presented in Supplementary Table S1.

### Adverse Effects

Due to the effects of omega-3 PUFAs on haemostasis, there is a potential risk of bleeding.

With regards to a risk of possible bleeding as an adverse effect of omega-3 PUFAs supplementation (individual or used as an enhancement of standard therapy), currently no clinically relevant indications have been suggested [75]. It has been shown that there is no higher risk of bleeding as a post-surgical complication in patients taking only fish oil and no additional anticoagulants [76]. Interestingly, there is a study on the possible occurrence of arrhythmias as an effect of supplementation. The authors suggest a need to precisely document the occurrence of arrhythmias in both present and future trials in order to assess the possible adverse effects of supplementation on nonfatal arrhythmias [77].

## 6. Novel Perspectives of Omega-3 PUFAs

Despite some controversies regarding the cardiovascular effects of omega-3 PUFAs, mainly regarding their role in dyslipidaemias and ventricular arrhythmias [16,50,67], there is increasing evidence of their anti-thrombotic properties. The work of Bonutti et al. described the synergistic anti-thrombotic effect of a combined therapy using aspirin and fish oil in the prevention of venous thromboembolism (VTE) after surgery in patients suffering from primary total knee arthroplasty. The results of this study suggest that the simultaneous application of aspirin and fish oil can be a good approach for the prevention of thrombosis in patients after surgery [78]. Recently, the anti-thrombotic and anti-inflammatory activity of omega-3 PUFAs has been observed in patients at risk of VTE [79,80]. According to the study of Isaksen et al., a high intake of marine omega-3 PUFAs in the diet was associated with a reduced risk of VTE after unprovoked index events [81]. In another study, it was demonstrated that omega-3 PUFAs consumption leads to a lower risk of both VTE and recurrent VTE [82]. There is also a discussion of the ability of omega-3 PUFAs to decrease the risk of deep-vein thrombosis and pulmonary embolism after surgical procedures [80,83].

Currently, there are a few publications analysing the antithrombotic properties of omega-3 PUFAs in patients hospitalized due to COVID-19 [84,85]. It is known that coagulopathy is frequently observed in many—especially severe—cases of COVID-19 and manifests as disseminated intravascular coagulation (DIC). The International Society of Thrombosis and Haemostasis (ISTH) has suggested the introduction of a new category for the early phase of sepsis-associated disseminated intravascular coagulation, called “sepsis-induced coagulopathy” (SIC) [85]. Currently, there are no results clearly suggesting the beneficial effects of omega-3 PUFAs in COVID-19 patients. However, it seems likely that the dietary intake of omega-3 PUFAs and/or their metabolites can exert positive effects contributing to the prevention and management of cardiovascular and thrombotic complications in patients suffering from COVID-19. Omega-3 PUFAs can affect and modulate some of the adverse effects of the immune overresponse, limit coagulopathy, and affect cell signalling and gene expression [86]. Omega-3 PUFAs are well known to have antithrombotic, anti-inflammatory, and pro-resolving properties that can yield positive effects in COVID-19 patients [87]. Since beneficial effects of fish oil/omega-3 PUFAs in limiting thrombosis have been confirmed in many disorders and clinical settings, it now appears logical to investigate the potential role of fish oil/omega-3 PUFAs supplementation as an adjunct to pharmacotherapy in COVID-19 patients at risk of vascular thrombotic complications [84].

## 7. Concluding Remarks

In the presence of some contradictory results, we suggest a novel potential use of fish oil. One of the aims of this paper was to indicate a new possibility—the support of a traditional anticoagulant treatment with the supplementation of EPA and DHA contained in fish oil. We believe it is a promising but still insufficiently explored research field. It seems likely that EPA and DHA being the sources of immunomodulatory metabolites (suppressing the inflammatory response) [88] could increase the effectiveness of antiplatelet drugs [89].

In our opinion, the recommended duration of the supplementation highly depends on the type and objective of the study and obviously on the group of the patients/subjects. In this paper, we have discussed both studies investigating the mechanisms of action as well studies focused on determining beneficial effects on health. For EPA and DHA, it is necessary to obtain maximum metabolite concentrations (2–4 h) [88], as well as to determine the duration of exposure for specific research subjects. The supplementation time among healthy adults should be in the range of 15 days [59] to 6 weeks [90].

While assessing the effect of fish oil supplementation on hemostatic parameters, platelet viability (10 days) and short half-lives of clotting factors (up to 5 days) should be taken into consideration. Supplementation in this case (minimum 4 g per day) should last no less than 2 weeks. On the other hand, assessing the effect of EPA/DHA metabolites on vascular endothelium, we recommend supplementation to last 6 weeks.

For the analysis of omega-3 PUFAs effects on health, the time of the supplementation should be significantly longer. Clinical trials ranged on average from 4 weeks to 6 years of observation (with a 90-day intervention), and in some cases the observation lasted even 10 years [81]. An analysis of the available literature suggests that the minimum duration of supplementation should be no less than 6 weeks. 

With regards to a dose of omega-3 PUFAs required for the supplementation, it is assumed that fish oil effectively inhibits platelet adhesion, obtaining a maximum beneficial effect, with a daily amount of EPA of about 6 g [28,54,59]. Based on the results of the REDUCE-IT study (including icosapent ethyl at 4 g/day) [60] and the efficacy of a single dose of 4.5 g/day [88], we assume that supplementation should be no lower than 4 g/day. In the study by Souza et al., plasma specialized pro-resolving mediator (SPM) levels were analyzed in healthy volunteers. An increase in SPM concentration was observed as a function of dose and duration of supplementation, obtaining a peak within 2–4 h after supplementation. A significant supplementation effect on the organism’s immune response was noted at a dose level of 4.5 g [88]. The importance of the proper dose of omega-3 PUFAs is also evident in the light of the recent meta-analysis by Bernasconi et al., who demonstrated that supplementation with omega-3 PUFAs resulted in a decreased risk of myocardial infarction. Such a trend was not observed in the case of cardiovascular events. In both cases, these observations were dependent on the supplemented doses of EPA and DHA that ranged from 400 to 5500 mg/day (data from 40 studies). The risk of myocardial infarction was significantly reduced by 9% for each additional dose of 1 g per day. Omega-3 PUFAs seem to be much more effective in a population with higher cardiac risk [51]. 

In general, in our opinion, patients being on a Western diet characterized by generally lower omega-3 PUFAs intake may require a higher dose of PUFAs (>4 g/day EPHA) and 6-week supplementation in order to obtain a significant outcome of omega-3 PUFAs effects on haemostasis.

## Figures and Tables

**Table 1:** Potential mechanisms of antiplatelet effects of EPA and DHA.

Number in Figure 2	Haemostasis Component	Potential Mechanism	Effect	Strength of Evidence	Reference
1	Prothrombinase complex (factors Va, Xa, II) on the surface of blood platelets	Decrease in cell membrane fluidity resulting in poor availability of procoagulant phospholipids (phosphatidylserine)	Slower conversion of prothrombin into thrombin; inhibition of coagulation cascade and platelet aggregation	Low	Larson [26]
1a	Ionotropic calcium channels in plasma membrane	Decrease in cell membrane fluidity resulting in reduced influx of Ca^2+^	Inhibition of blood platelets activation	Low	Kacik [31]
2	Synthesis of TxA_2_	Competition between AA and EPA for enzymes essential for further metabolic steps	Inhibition of TxA_2_ synthesis leading to a production of TxA_3_ having weak pro-aggregatory properties; inhibition of TXA_2_-dependent platelet activation	High	DeFilippis Adili, Bäck [28,32,33]
2a	Synthesis of TxA_2_	Metabolite of EPA–resolvin binding to TxA_2_ receptor	Inhibition of TXA_2_-dependent platelet activation	Moderate	Sheikh, Bäck [33,34]
3	Receptor for ADP and receptor for thrombin	Metabolite of EPA–resolvin binding to receptors for ADP and thrombin	Inhibition of TXA_2_-and thrombin-dependent platelet activation	Moderate	Fredman, Dona [30,35]
4	Receptor GPVI (collagen receptor)	Blocking of the receptor (detailed mechanism unknown) or inhibition of platelet reactivity in a glycoprotein VI-dependent manner via activation of protein kinase A	Inhibition of collagen-dependent platelet activation	Low	Larson [36] Yamaguchi [8]

**Table 2:** Potential mechanisms of effects of EPA and DHA on endothelium.

Number in Figure 3	Haemostasis Component	Potential Mechanism	Effect	Strength of Evidence	Reference
1	Endothelial synthase of nitric oxide (eNOS)	Higher production of NO	Vasodilatation effect	High	Łacheta, Yamagata [39,40]
2	Adhesive receptors	Decrease of expression of adhesive molecules (VCAM, ICAM); reduced expression of tumor necrosis factor alpha (TNF-α) and PAI-1		High	Wang, Liu [41,42]
2a	Protein synthesis	Decreased synthesis of endothelin-1	Anticoagulation effects	Moderate	Yamagata [40]
3	Receptor for t-PA	Higher level of t-PA	Fibrinolytic effects	Low	Din [43]
4	Receptor for oxLDL	Regulation of expression of the receptor for oxLDL	Inhibition of formation of oxidized LDL	High	Chen [44]
5	Inhibition of microparticles formation	Lower expression of tissue factor	Reduction of oxidative stress, inhibition of thrombin generation	Low	Qureshi [45]

## Supplementary Materials

The following are available online at https://www.mdpi.com/1422-0067/22/5/2394/s1.
