# Supplementary material for: Effects of Omega-3 Polyunsaturated Fatty Acids and Their Metabolites on Haemostasis—Current Perspectives in Cardiovascular Disease
Source: Int J Mol Sci. 2021 Feb 27;22(5):2394. doi: 10.3390/ijms22052394 (PMC7957531; doi:10.3390/ijms22052394)
Supplement: Supplementary file 1 [file ijms-22-02394-s001.pdf]

Table S1. A list of epidemiological and clinical studies on effects of fish oil omega-3 on haemostasis

Author and year	Description of the study, number of participants	Source and dose of omega-3	Length of supplementation or observation	Outcome	Commentary
{Dyerberg, 1979 #1072}	Observational, cross-section study. Greenland Inuits, (10 women, 14 men). Control: 21 healthy age and sex matched Danes	Typical Inuits dietary habits. Typical Danish dietary habits.	Not applicable	Inuits had significantly longer bleeding-time	Lack of differences in blood platelet reactivity and coagulation parameters
{Rogers, 1987 #1071}	A double blind randomised controlled trial. 60 male volunteers	Fish oil supplement (2.9 g EPA in the first week followed by 1.6 g daily for the remainder of the period)	3-6 weeks	No effect of fish oil supplementation on platelet aggregation, thrombin time, fibrinogen level, platelet factor 4 level	
{SIMONSEN, 1987 #1070}	Dietetic questionnaire study. Two randomly selected 20 healthy male subjects from inland vs. coastal population.	Local dietary habits. Fish consumption was 2.5-fold higher in the coastal group	Not applicable	Subjects from the coastal area had platelets with increased sensitivity to collagen	The daily consumption of omega-3 PUFAs was 0.6 g in the inland and 2.2 g in the coastal groups
{Skeaff, 1988 #1068}	Crossover study. 8 healthy male volunteers	Fish oil (3.7 g of EPA and 2.4 g of DHA per day)	6 weeks followed by a 6 week "wash-out" period (no fish oil was consumed)	Dietary fish oil supplementation resulted in decreased maximum aggregation responses to collagen and platelet-activating factor (PAF) by 50.1% and 27.2%, respectively	Dietary fish oil consumption significantly diminishes washed platelet responses to collagen and PAF.
{Bach, 1989 #1073}	Double-blind, placebo-controlled study. 30 healthy subjects	1.26 or 2.52 g omega-3 fatty acids<br>The placebo capsules contained the neutral oil	5 weeks	The spontaneous platelet aggregation was significantly reduced in the fish oil group but also in the placebo group	No significant differences occurred at any time between the fish oil and placebo group.
{Lox, 1990 #1090}	Follow-up interventional study, 9 healthy males	3 g of fish oil (900 mg omega-3 fatty acids, including 540 mg EPA mg and 360 mg DHA)	30 days	No effect on platelet aggregation. Coagulation factors activity was significantly affected by the treatment.	Low level ingestion of marine fish oil has a beneficial effect on coagulation.
{Malle, 1991 #1069}	Follow-up interventional study, 15 healthy normolipaemic male subjects	Fish oil capsules (3.8 g EPA and 2.9 g DHA/d)	3 measurements points: before supplementation;	Supplementation resulted in a decrease of collagen- or	

			after 6 weeks supplementation; after 4 weeks without supplementation	thrombin-induced platelet aggregation by 45% and a reduction of collagen- or thrombin-stimulated TXB, generation by 40%. Four more weeks without dietary supplementation restored the pretreatment values of platelet aggregability	
{Li, 1991 #90}	Dose-response study, 15 normal, healthy volunteers (11 women, 4 men)	Fish oil capsules (equivalent to 3, 6, or 9 g EPA/day)	21 days	Maximal inhibitory activity was observed at the dose of 6 g EPA/day The reduction of platelet adhesion was 85.5%.	Platelet adhesion to fibrinogen demonstrated significant decrease in response to fish oil administration (3 6 g EPA/day vs. 6 g EPA/day)
{Andrioli, 1999 #1119}	Randomised study, 60 volunteers (30 men and 30 women)	3 groups taking 20 ml (equivalent to 0.3 g ω-6, 3.6 g ω-3; ω-6/ω-3 ratio 0.1) per day of a fish oil supplement, or 25 g (equivalent to 1.5 g ω-6, 0.5 g ω-3; ω-6/ω-3 ratio 3) per day and soy lecithin supplement group.	15 days	Fish oil group showed a significant reduction in stimulated adhesion (with ADP or thrombin)<br>In the soy lecithin group, platelet adhesion was increased in all test conditions	The authors have suggested that the ω-6/ω-3 ratio is a determinant of platelet adhesion.
{Sakamoto, 2000 #1108}	Fibrinolysis generated by 1 h physical load in 30 male subjects<br>Measurements of haemostatic parameters before the load ad 1 h after load.	EPA tablets (1.125 g/day)	2 weeks	Plasmin-α2 plasmin inhibitor (PIC) complex level was decreased by 16.7%, and the thrombin-antithrombin III complex (TAT) level was increased by 75.4%;<br>D-dimer level was significantly decreased by 24%	Low dose of EPA significantly decreased the fibrinolytic activity and increased the coagulation activity 1 h after a physical load.
{Nordøy, 2003 #1111}	Double-blind, placebo-controlled parallel study, 42 patients with combined dyslipidaemia treated with atorvastatin	The patients were randomized into two groups receiving 2 g/day ω-3 fatty acids (puried EPA) 45% (DHA) [39%] capsules or placebo (2 g/ d corn oil)	5 weeks	The supplementation reduced the postprandial level of FVIIa, FVII:C and FVII-Ag	Patients with hyperlipemia are at risk for activation of the coagulation system, particularly during postprandial lipaemia. This activation can be reduced by statins and omega-3 PUFAs

{Lindman, 2004 #1075}	The trial had a 2x2 factorial design, 219 subjects were randomly allocated to the groups	Two capsules twice daily corresponding to a daily intake of either 2.4 g ω-3 fatty acids or 2.4 g corn oil were given	6 months	Significant reductions were observed in the diet intervention group for FVII:C, FVII-Ag, and FVIIa	The reduction of FVII levels after diet intervention can be important for the reduction of the risk of CVD
{Vanschoonbeek, 2004 #1118}	Follow-up interventional study, 25 healthy male subjects with borderline overweight (body mass index 29.0±2.5 kg/m2)	Fish oil (3.0 g omega-3 PUFAs/day)	4 weeks	Reduced plasma levels of the vitamin K-independent coagulation factor V (significant) and of fibrinogen (borderline significant), but not of the vitamin K-dependent factors, prothrombin, factor VII, and factor X.<br>Fibrinogen antigen level was significantly reduced.<br>Flow cytometric analysis indicated that fish oil intake significantly lowered SFLLRN induced activation of αIIbβ3 integrin. In some, but not in all, subjects, fish oil intake resulted in a decreased thrombin generation	Dietary omega-3 PUFAs induced a hypocoagulant, vitamin K-independent effect, the degree of which may depend on fibrinogen level
{Del Turco, 2008 #96}	46 post-myocardial infarction patients were randomly allocated in two groups.	5.2 g of n-3 fatty acids daily capsules (n=23) or an olive oil placebo (n=23)	12 weeks	Total microparticle tissue. factor-procoagulant activity was reduced in the n-3 fatty acid group	Procoagulant potential are decreased after ω-3 fatty acid administration. ω-3 fatty acids exert beneficial effects after myocardial infarction in humans.
{Gajos, 2011 #336}	Prospective, double-blind, placebo-controlled, randomized study in 54 patients undergoing PCI	1 g/day ω-3 PUFA capsule (containing ethyl esters of 460 mg of EPA and 380 mg of DHA) (n=30) or placebo (n=24)	1 month	ω-3 PUFA compared with placebo was associated with 15.3% higher fibrin clot permeability indicating larger pores in the fibrin network indicating increased susceptibility to fibrinolysis.	These findings indicate novel antithrombotic effects of by ω-3 PUFA in humans.
{Larson, 2011 #1033}	Open-label, sequential therapy study. Subjects served as their own controls. 30 healthy volunteers	Blood was drawn: Day 1 (control), Day 2 (one day after a standard therapeutic 650 mg	28 days	EPA and DHA significantly decreased platelet reactivity (increased closure time of the	The lowered platelet response to collagen may account for some of the

		dose of aspirin), Day 29 (after 28 days of 4 g/d of ω-3 capsules delivering 1.86 g/d EPA and 1.5 g/d DHA) and Day30 (after one day of combined ω-3 capsules and 650 mg aspirin treatment).		collagen/epinephrine cartridge) after aspirin treatment.	cardioprotective benefits provided by DHA and EPA.
{Phang, 2012 #1080}	A placebo-controlled trial. 15 males and 15 females	Single dose of 2×1-g capsules containing either placebo (sunola oil) or EPA-rich oil providing 1 g EPA with an EPA/DHA ratio of 5:1 or DHA-rich oil	24 hours after supplementation	EPA and DHA reduced platelet aggregation at 24 h postsupplementation relative to placebo. Females showed significantly reduced platelet aggregation but not MP activity after DHA only.	EPA and DHA exert gender-dependent effects on platelet aggregation and platelet MP activity, but not on MP levels. With respect to thrombotic disease risk, males may benefit more from EPA supplementation.
{McEwen, 2013 #238}	Cohort study. 40 healthy subjects and 16 patients with CVD	PUFA capsule (DHA 260 mg and EPA 60 mg) twice daily	4 weeks	In healthy subjects, omega-3 PUFA significantly reduced ADP–induced and adrenaline-induced platelet aggregation and P-selectin expression	640 mg omega-3 PUFA reduced measures of platelet aggregation and activation in healthy subjects but effects were less evident in patients with existing CVD
{McEwen, 2015 #76}	Cohort study. 40 healthy subjects and 16 patients with CVD – Takie same grupy w obu badaniach jak powyżej?	PUFA capsule (DHA 260 mg and EPA 60 mg) twice a day	4 weeks	Four-week omega-3 PUFA supplementation in healthy subject fibrin generation.	There was a greater effect on fibrin generation in healthy subjects compared with those with CVD
{Bonutti, 2017 #1096}	Prospective cohort study. 850 patients with a history of thromboembolic disease undergoing total knee arthroplasty.	250 patients received a 4-week dose of rivaroxaban<br>300 patients received a 90-day dose of 325 mg aspirin<br>300 patients received 325 mg aspirin and 1 g fish oil.	90-day intervention<br>6 years observation	Patients who received aspirin and fish oil demonstrated significantly lower risk for thromboembolic events when compared to the aspirin and pulsatile stocking group. When compared to the rivaroxaban cohort, the ORs did not differ significantly. When compared to rivaroxaban, the fish oil and aspirin cohort demonstrated significantly lower incidence of bleeding episodes	This study demonstrated the potentially synergistic anti-thromboembolic effect of aspirin and fish oil in the prevention of post-operative venous thromboembolism in total knee arthroplasty patients

{Corrocher, 2017 #1094}	Randomised study. 60 volunteers (30 men and 30 women), allocated into three groups	fish oil: 20 ml (equivalent to 0.3 g ω-6, 3.6 g ω -3; ω -6/ ω -3 ratio 0.1), 25 g (equivalent to 1.5 g ω -6, 0.5 g ω -3; ω -6/ ω -3 ratio 3), soy lecithin supplement,	15 days	Fish oil group showed a significant reduction in platelet adhesion induced with ADP and thrombin.<br>In the soy lecithin group, platelet adhesion was increased in all test conditions	inhibitory effect of fish oil rich in ω -3 fatty acids on stimulated human platelet adhesiveness and a stimulatory effect of soy lecithin rich in ω -6 fatty acids on resting and stimulated adhesion. They suggest moreover that the ω -6/ω-3 ratio is a determinant of platelet adhesion.
{Bagge, 2018 #403}	Brak typu badania. 12 Caucasian volunteers (5 women and 7 men)	Fish oil capsules, 630 mg of omega-3 (EPA – 300 mg, DHA – 200 mg) twice daily	10 days	Omega-3 fatty acids had no effect on platelet reactivity and clot formation.	study on healthy volunteers, no effects of high doses of omega-3 fatty acids could be demonstrated after 10 days of intake, either on coagulation or platelet function, when measured using Sonoclot, MEA and a microfluidic model.
{Isaksen, 2019 #79}	Single-center cohort study with repeated health surveys. 595 patients with incident VTE	Self-administered complete food frequency questionnaires assessing fish intake and use of fish oil supplements<br>n-3 PUFAs (g/wk)<br>T1 (< 8.19)<br>T2 (8.19–29.1)<br>T3 (> 29.1)	10-year observation	High intake of n-3 PUFAs was associated with a significant 49% lower risk of recurrence unprovoked VTE	A high dietary intake of marine n-3 PUFAs was associated with lower risk of recurrent VTE after unprovoked index events, DVT, and in cancer-free patients.
{Bagger, 2020 #5}	Observational non-randomized, non-blinded screening study. 10 healthy male volunteers	Two capsules of Omega-3 fish oil (1260mg/day) Ile było EPA? (EPA), 400 mg docosahexaenoic acid (DHA), 60 mg docosapentaenoic acid (DPA) and 200 mg of other Omega-3.	5 days	No significant changes in the ADP, TRAP or ASPI-assays after Omega-3 intake.<br>No additive or synergistic platelet inhibitory effect of 5 days peroral ingestion of Omega-3 and ASA (in vitro) was found.	
{Zheng, 2020 #50}	Randomised study. 452 patients with proximal femoral fractures were	Fish oil 1000 mg (mamy skład?)	30 days	Daily supplementation of omega-3 fatty acids decreases the risk of pulmonary embolism as	

	randomly assigned to receive either omega-3 fatty acids or placebo.		well as symptomatic deep vein thrombosis, after surgery among elderly patients with proximal femoral fractures, without causing elevated risk of bleeding episodes	
